# The Adaptation of Means in the Prevention of Disease

**Published:** 1906-09

**Authors:** D. S. Davies

**Affiliations:** Medical Officer of Health, Bristol


"*Scire est nescire, nisi id me*

*Scire alius sciret.*"

INDEX THROUGH

SEPTEMBER, 1906.

### THE ADAPTATION OF MEANS IN THE PREVENTION OF DISEASE.

### AN ESSAY TO ILLUSTRATE THE IMPORTANCE TOWARDS PREVENTION OF A CAREFUL STUDY OF THE LIFE HISTORY OF INDIVIDUAL DISEASES.

The Presidential Address read at the Annual Meeting of the Bath and Bristol Branch of the British Medical Association, May 30th, 1906.

BY

D. S. Davies, M.D. Lond., D.P.H. Cantab.
*Medical Officer of Health, Bristol.*

In a previous address which I was privileged to deliver before the Bristol Medico-Chirurgical Society,[1] I endeavoured to point out the inter-relation of public health with curative medicine and surgery, and also to review the problems of communicable diseases and their causation; first, from the ancient standpoint, which consisted in a superstitious dread of epidemic disease as a mysterious, inexplicable and unavoidable visitation, and, secondly, from the modern view, in which a careful study of the life-history of causal parasitic organisms, and also of the conditions promoting or assisting their distribution, plays so

[1] *Bristol M.-Chir. J.*, 1900, xviii. 289.

important a part, and tends to remove year by year much of the obscurity which formerly surrounded these diseases. I tried to show that epidemics may not now be considered as inchoate outbursts, showered by the opening of Pandora's box upon the devoted heads of mankind; for Hope which remained imprisoned has blossomed, and is in many directions bearing the fruit of success, through the application of science, that is of accurate knowledge, to the study and treatment of disease, an application which, as was appositely pointed out by my distinguished predecessor in this chair, constitutes the essential standpoint of the practice of modern medicine and surgery, of which Public Health is but a part. I pointed out that epidemics must be considered as the outcome of single cases or groups of cases, increasing along definite lines, which perfect knowledge might always trace back to the initial cases, and that the checking of these lines of spread, together with supplementary measures designed to make the conditions of life unfavourable to the parasitic habit, forms a large part of modern Public Health work.

I then discussed, following Leuckart and Payne, the origin of parasitic disease-organisms from free-living species which have adopted the parasitic habit under favourable environment, and remain subject to the three parasitic laws of "entrance," "migration," and "exit," whereby they obtain increased facilities of continued existence as a species.

Finally, I considered the interacting causes, political, social and medical, which were at work during the eighteenth century, and led up to the modern conditions of life and labour; new social conditions, under which the struggle with cholera had to be waged, unsuccessfully at first, but in time resulting in the beginning of modern Public Health work, which means far more than the mere care about communicable disease, though in many ways arising out of and bound up with its prevalence, and which has for its wider aims the care of every class of the community—aims not yet fully realised, but full of promise for the future.

It may not be entirely void of interest and benefit if I attempt to review, however briefly, and, I fear, imperfectly, the progress in some few branches of discovery relating to communicable disease, and the changes in medical thought which have accompanied advance in knowledge.[1]

The brilliant series of discoveries demonstrating the causal organisms of disease, which commenced in 1880, had been led up to and rendered possible by the painstaking and indefatigable work of numerous observers scattered over a period of some two centuries. It was Athanasius Kircher who in 1671 started, however imperfectly, a "Pathologia Animata," while Leeuwenhoek in 1718 enlarged the possibilities of microscopic observation; the shrewd guesses of Varro, of Lancisi and of Linnæus were followed up by Marcus Antonius Plenciz, who insisted upon the specific character of the infective agents in disease, and of the causal agents in putrefaction.

It was not, however, till the years following 1831 that any substantial advance was made. Otto Friedrich Müller, of Copenhagen, had indeed attempted to classify the minute organisms he observed, but Needham intervened with his theory of spontaneous generation, in turn destroyed by Bonnet's acute criticism and Spallanzani's crucial experiments, completed and confirmed by Schulze. In 1854 Schrœder and Van Dusch made further advances, and were the first to utilise cotton wool filtration of air; Tyndall, Schwann and other workers laboured at home or abroad, until at last the belief arose that if bacteria are once quite destroyed in any medium, no other organism can rise from their ashes, and the medium remains sterile until reinfected from without. Harvey's dictum of "omne vivum ex ovo" was thus becoming accepted in a far wider sense than he could have intended.

Meanwhile attempts were in train to improve the classification of Müller; in 1854 Cohn insisted on the plant nature of micro-organisms, and in 1857 Nægeli collected all the forms then known under the heading Schizomycetes or fission-fungi.

To go back a little. In 1837 Cagniard, Latour and Schwann asserted the connection of yeast cells, originally described by Leeuwenhoek, with fermentation; and in the same year Bassi associated certain spores found on or within the bodies of affected

[1] Cf. Sims Woodhead, *Bacteria* (Contemporary Science Series).

silkworms with the silkworm disease, the first fully-described instance of a fungus parasite giving rise to disease in an animal, although fungus disease of plants had been long recognised. Henle, in 1840, as a result of these researches, believed that the cause of "miasmatic," "infective," and "contagious" diseases must be looked for in living fungi, or other minute living organisms, but, in spite of much attention devoted to the subject, no definite proof was then obtained. At this stage (1857) Pasteur took up the fermentation work initiated by Cagniard, Latour and Schwann, and later he took up the study of silkworm disease (1862), and, in brief, he demonstrated that certain specific organisms endowed with definite morphological and physiological characters gave rise by their presence to specific and characteristic diseases; he had, in effect, extricated the theory of contagium vivum from a condition of chaos, and placed it, in regard to certain diseases, in a definite and certain form. The most important part of the work that followed consisted in the attempts to obtain pure cultures of micro-organisms, led up to by Klebs and Brefeld's gelatine method, perfected by Koch.

The time was thus becoming ripe for important discoveries as to the intimate nature of disease causation, but, while events were maturing towards this end, organised effort had already begun in the direction of the wider measures of Public Health work, dealing not alone with the communicable diseases, but with all those conditions of life which, by giving no sufficient opportunity to the mass of the people for a healthy existence either in their homes or at work, prejudiced not only their physical but their moral well-being. This awakening, and the prompt inquiry and action which the pioneers of the movement took care should follow, happened when cholera had already, in 1831 and the following years, provided a definite and a terrible object lesson. The commencement of Registration of Deaths supplied important data, and the early Public Health Acts followed in due course.

It was inevitable that opinion, formed under such auspices, and guided by revelation of the gross conditions which were almost universally found to exist, should have mainly concerned itself about filth conditions, and have mainly advocated "municipal cleanliness" not only as a remedy for the existing evils, but, by a process of wide generalisation, as the remedy for the prevention of all epidemic disease.

In every sense it was well chosen as a watchword, and the direct and indirect benefits that have accrued from its steady prosecution have been neither few nor insignificant. Possibly no other line of action could have resulted in so much progressive improvement of the general conditions of life and work, in so great improvement of the national health, in so large a reduction of the general mortality. It also happened that the diseases which were during that period either persistently, as typhus and enteric fever, or intermittently, as cholera, levying a heavy and preventable toll of human life, were peculiarly susceptible to the influences of general sanitary improvement; for the natural history of the intestinal group of diseases is such that they find in polluted soil and water the necessary conditions for the continuance of their parasitic species, while typhus or jail fever can, on the other hand, only thrive and spread where overcrowding, deficient ventilation and neglect permit it to smoulder and persist, as it will notably do where overcrowding of persons in houses accompanies overcrowding of houses on sites.

But it was soon to be discovered that certain of the infective diseases are not so directly influenced by municipal cleanliness. This by itself is ineffectual in the case of many infective diseases in which the handing on of infection is immediate and personal —overcrowding, filth of habitation or of person, seem in such cases to have a very secondary influence, consisting mainly in this, that sections of communities affecting these habits show a gregariousness and a lack of precaution beyond the ordinary, and thus afford the close contact and other similar opportunities necessary for transmission. I do not even know that poverty or its concomitants necessarily connote increased susceptibility to infection, for susceptibility often seems to be an individual and possibly an inherited tendency, largely or wholly independent of environment. Be this as it may, there can be no doubt that while the securing of municipal and personal cleanliness was a praiseworthy and a practical ideal, it was by itself an insufficient bar to the dissemination of many of the infective group of diseases.

The scientific study of disease origin to which I have already briefly referred, was to bear notable fruit in the years following the mid-century. In 1863 the relation of the anthrax bacillus to splenic fever had been established, but all previous work was eclipsed by the series of remarkable discoveries that centred round the years 1880 to 1885. The determination of the enteric fever bacillus by Eberth in 1880 headed the series, followed by the joint exposition of diphtheria by Klebs and Löffler in 1883–4, and by the demonstration in 1884 of the spirillum of cholera by Koch, whose culture methods gave the opportunity to bacteriological research of completing the demonstration of causal relationship between the organism and the disease. Ten years later Kitasato demonstrated the cause of plague to be a bacillus, and, though smallpox and certain other communicable fevers have presented greater difficulties in the determination of their actual causal organisms, enough had been achieved to profoundly affect not only medical but lay opinion.

The immediate result was to establish a second period, in which the original generalisations in regard to "filth" causation of disease were replaced by generalisations equally wide based upon the now acknowledged parasitic and transmissible character of the infective fevers. It seemed plausible to assert that, the cause and its immediate result, disease, being known, isolation of the cases and destruction of residual infection should suffice to blot out such disease. The control and elimination of the communicable fevers appeared to have been made simple; all such diseases were now classed as "preventable," and immediate and complete control was looked for. Stimulus was given to the provision of isolation hospitals, disinfection was systematically studied and applied; but as the years progressed it was found, to take for example the case of scarlet fever, that in large centres of population the decline of fever mortality does not necessarily follow the provision of isolation hospitals, and often appears to be entirely independent of measures of prevention. The value of isolation hospital accommodation has thus come to be seriously questioned, and it is urged that if isolation fails to control disease, it is useless and a waste of money to build isolation hospitals. The question is a difficult one, but it is worth some careful consideration.

In considering infective disease the inquirer must take into account, not only the causal agent, but the soil on which it grows, and, in fact, the whole natural history of the disease in question, which includes the questions of individual resistance, virulence of the epidemic wave, social and school conditions as predisposing to readiness of infection, protection by recent outbreaks, "mediate" (or indirect) as well as "immediate" (or direct) personal infection.

Disease varies so widely in its natural history that we can no longer safely generalise on the whole group, but must attempt to gain a complete knowledge of the natural history of each disease, on which to base appropriate preventive measures.

I have elsewhere stated that the "essential conditions" under which isolation of acute infective fevers should succeed in preventing extension are:—

1. The patient must not have handed on his infection to any other person before his seclusion commenced.
2. The patient must not have left behind any infected material, or, if he has, this must be rendered sterile.
3. The patient must be kept perfectly secluded, through the whole of his illness and convalescence, from direct or indirect communication of his disease to others.
4. The patient must have perfectly recovered, and must be cleansed both from his own and from any superadded infection which he may "carry" out, before he is discharged from seclusion.

Certain diseases, for example, smallpox and typhus fever, have shown themselves to be fully amenable to preventive measures, amongst which hospital isolation has been one necessary factor. Let us take smallpox, as at present met with amongst partially vaccinated communities, for one example, and see in what way its natural history conduces towards the success of isolation (including of course the ordinary accompanying disinfection processes).

The causal agent of smallpox is so highly organised that it is selective in regard to the soil on which it grows, and it will grow neither on an unsuitable nor an exhausted soil. Also we can render the soil of contacts unsuitable by inducing vaccinia. If the soil is naturally unsuitable, the person exposed to infection neither *contracts* smallpox nor *carries* it; the infection simply dies out. If, on the other hand, the soil is suitable, the disease runs its acute course, after which the soil becomes exhausted and the disease cannot, and never does, persist in the patient. So we get no chronic cases, and no "return" cases. It may therefore be taken as an axiom, that a patient either definitely has or has not smallpox at any particular time; he never merely carries it on his living tissues in an inert, but, to others, a potentially infective form. Also this disease, though of high infectivity, is, I believe, only infective at short range (as, for example, to bed-contacts) for the first two days before the distinctive eruption appears. Owing to these features in its natural history, the four essential conditions can, with the exercise of some patience and no little care, be readily fulfilled, and the methods and value of hospital isolation in the checking of the offshoots from primary introductions of disease into a district can, in the case of this disease, be usefully demonstrated. In this city we have a record of twelve years' unbroken success in limiting and quickly obliterating a long series of introductions. *Absit omen!*

We may contrast with this disease diphtheria, whose natural history renders it much less amenable to hospital isolation than smallpox, although it resembles it in being, as I believe, dependent on *personal* and not on *place* infection.

The parasitic fungus of diphtheria appears, however, to be more lowly in organisation, it is certainly not selective as to its soil. Most or all human soils prove suitable. It will grow in some form or other upon the mucous surface of the palate, in the nose, the ear, or on the raw skin surfaces of probably nine-tenths of all children under twelve, and somewhat less freely upon older persons. Of these nine-tenths who receive the organism, retain and grow it (*i.e.* become affected), only about one-half are susceptible to intoxication by the alkaloidal poison-product of the fungus (*i.e.* become infected and suffer from clinical diphtheria to an extent to be inconvenienced by it), the rest suffer either from insignificant symptoms, or in very many cases show no symptoms at all, but merely *carry* the fungus; themselves naturally immune, they form a constant menace to others. Further, an exhausted soil forms no bar to further growth; the patient who has passed through the clinical disease recovers, but the fungus, though no longer producing toxic effects on the patient, may continue to grow, and if it has gained access to the recesses of the nasal cavity, to the antrum, or to the frontal sinuses, may apparently, upon occasion, fortunately not often, become chronic, and persist for months or years.

A disease with such a natural history obviously presents unusual difficulties in securing the essential conditions of success in isolation. Indeed, isolation of obvious clinical cases alone would prove quite inoperative in checking the progress of an epidemic, for the unmarked cases left at home may far exceed the number moved to hospital; disinfection as a general measure would prove equally inoperative, as it would not touch the harbourers of personal infection. It is obvious that special methods with the main object of detecting and dealing with *minimal* and with *carrier* cases must be adopted—such methods as, following American experience, we have attempted to apply in this city since 1895, with varying success under varying phases of bacillary invasion, and in districts of varying amenability to discipline, the poorest districts being by no means the worst. But hospital isolation, though no longer to be considered as a fetish charged with the immediate extinction of the disease, is still an invaluable and indispensable adjunct to such measures as I have described, based on the known natural history of the disease.

Similarly typhus fever, which, I may remind you, was once fatally endemic in Bristol, has proved amenable to isolation, allied with suitable adjuvant measures, whilst scarlet fever, resembling diphtheria in many of its habits as to persistence, has proved refractory. I am not sure, however, that the present very favourable type of scarlet fever, which presents a case mortality of some 3 per cent., is not due in great part to the judicious isolation of cases which in former days would have been left in crowded homes where aggregation and uncleanliness would promote the occurrence of septic forms. Removal to hospital of scarlet fever cases from an ordinary well-to-do home with a spare room, is both inadvisable and unnecessary. It is startling to realise that scarlet fever, which for the past sixteen years has only once caused as many as sixty deaths in the year, was responsible in the epidemic of 1869-70 for over nine hundred deaths.

Diphtheria has, during its progressive prevalence of late years in one district after another, taught us many lessons of incalculable value. The importance of the detection of *carrier cases* in this disease has been so fully recognised in this city, and I have received so much willing and invaluable co-operation from the medical profession in carrying out the necessary investigations and in applying the suitable preventive measures, that I gladly take this opportunity of acknowledging it, and of expressing my grateful thanks. The "filth theory," excellent towards the attainment of a cleanly life, is so inadequate and so ineffective when applied towards the control of a personally spread disease that it may prove a positive danger, and the practical acceptance by local practitioners of facts based upon a study of the natural history of the disease is evidence of real progress.

It is regrettable that the Medical Officer to the London Education Committee, in his Report for the year ending March, 1905, should have to report that these facts in the natural history of diphtheria are as yet unknown to practitioners, who are generally unaware that diphtheria may be a mild complaint, which cannot be clinically recognised, or that healthy contacts may be "carriers" of the complaint, and that there are London boroughs where the Sanitary Authority make no arrangement for bacteriological testing of cases, so that after the preliminary exclusion of scholars by the Education Medical Officer, who undertakes the primary bacteriological examination in the case of schools, the cases are often very badly or not at all followed up by the Sanitary Authorities.

**Minimal and Missed Cases.** The occurrence of mild cases of all infectious fevers has for many years been recognised in textbooks, and the term "ambulatory" typhoid concisely expresses both the mildness and the risk of a certain form of attack. But it is only of late years that the absolute importance of "minimal" and "missed" cases has been fully realised. If the infective fevers were invariably severe and clinically well marked, prevention would be greatly facilitated: the "missed" case occurs as a result of "minimal" cases, and these unrecognised cases are becoming known as a chief factor in the spread of disease. For reasons I have mentioned, smallpox is the disease in which it is possible to obtain most definite results, and it is to the steady recognition of this principle, and diligent personal search for "missed" cases, that success lasting through twelve years has been attained.

"**Reconstruction**" **of Cases.** Arising out of the "missed" case and an essential to success, comes the necessity for occasional "Reconstruction" of cases—for example, it has on more than one occasion happened that, following upon the introduction of smallpox, a series of resultant cases has occurred at the recognised incubation periods of twelve days; then a lull has occurred, apparent cessation of the outbreak for some weeks; suddenly a fresh case is reported. Upon comparing dates, it has been found that the number of intervening days between the fresh case and the last known case is an exact multiple of twelve days. Say it is twenty-four days, you may then be quite sure there is an intervening missed case not reported, and if the outbreak is to be checked, this case must be found and dealt with: this is not generally difficult, with care, and upon the picking up of this link, or the reconstruction of the missing case, depends success. If the reported case only is dealt with, an outbreak is inevitable.

Dr. Kerr, Medical Officer to the London Education Committee, has in the Report mentioned drawn some very definite conclusions as to the behaviour of diphtheria. He agrees with our local practice in believing that school closure in diphtheria outbreaks is rarely necessary, and is equivalent to a confession of failure, either from want of time or want of staff, as he finds that the measures he takes are now efficacious in immediately arresting the spread of the disease in 95 per cent. of the outbreaks. He has formulated an interesting law with regard to diphtheria outbreaks which probably holds true in regard to communities resembling London in having passed through some years of continuous diphtheria prevalence during which a large portion of the population has become partially immunised by minimal doses.

The law is that, as applied to London, it is rare to find a school class whose average age is under five or over eight spreading diphtheria. Classes at other ages than five to eight may be attacked, but the disease does not give rise to school outbreaks, and never in his experience has a class of average age under four or over ten done so.

Now this age period involving the tendency to spread (five to eight) does not coincide with the age period of greatest diphtheria incidence, which is from two to four years, with a maximum at four. And herein lies the probable explanation. At birth a transient passive immunity is possibly inherited; this wears off, leading to an increasing incidence up to four years of age; hence onwards an active acquired immunity makes itself felt, and between five and eight there is a rapid diminution of the incidence rate, while after eight years of age protection becomes sufficiently general to prevent notable extension. It will be noted that the years of the rapid decline of incidence are precisely those during which classes become foci of infection, as the active acquired immunity (acquired from minimal or sub-minimal does), though sufficient to prevent a marked attack at these ages, actually tends to the development of "carrier" cases, which are apt to be "missed," and may thus lead to serious extension.

In the rare event of a "carrier" appearing in a babies' class, most of the contacts will contract clinical attacks, and spread will be limited; while over the age of eight or ten slight attacks are less common, and classes of these ages do not present spreading outbreaks.

Dr. Kerr's law is apparently true with regard to London, but the exact behaviour of diphtheria in this respect would vary with the period of invasion, that is to say, judging from my experience, it would not apply during the first two to three years of invasion by virulent diphtheria after a quiescent period of years, but it would naturally come true as the population gradually becomes leavened with the disease.

It is therefore a point of considerable interest and importance, as it tends to systematise and make practical the methods of dealing with school diphtheria outbreaks, under certain known conditions.

It has, then, become sufficiently evident from experience with home diseases that the study of the life history of parasites, including the interaction between them and the living bodies on which they grow, is essential towards an intelligent application of preventive measures in disease; this is still more remarkably true with regard to many of the exotic diseases such as malaria and yellow fever. Indeed, Manson has well stated that the student of medicine must now be a naturalist before he can hope to become a scientific epidemiologist.

In regard to these two diseases, in particular, the light shed by inquiry into their natural history upon their habits and prevalence, and the aid thereby afforded to preventive medicine has been little short of marvellous, and has marked out a third era in the annals of scientific preventive medicine.

Although the connection of malaria with swamps and high temperatures, the danger of night exposure, the influence of trees and water in connection with the disease have long been known, and popular opinion has often vaguely associated the disease with the mosquito, and indeed certain observers, including King, Laveran, Koch and Pfeiffer, have suggested such relationship, no definite hypothesis was formulated till quite recently, when in 1894 Manson suggested that the extra-corporeal life of the protozoal parasite of malaria was spent in a particular species of mosquito, which thus acted as an intermediate host. Following out Manson's suggested line of research, Ross in the years 1895-7 found the parasite in the stomach wall of the mosquito, and, in the case of proteosoma of birds showed that it entered the salivary gland. Ross distinctly proved two things; first, that the extra-corporeal phase of the malaria parasite is passed in particular species of mosquito (differing for the malaria of man and that of birds); and, secondly, that the parasite is transferred from man to man, or from bird to bird by the distinctive mosquito. MacCallum proved in regard to halteridium, another blood parasite of birds, the impregnating function of the flagellum, and the remarkable facts in regard to the travelling vermicule. The Italian investigators, and Daniels and Koch, confirmed these various facts. Grassi not only did this, but determined the effective species of anopheles for Italy.

Finally, the successful experiment of the malaria hut in the Roman Campagna in 1900, in which exclusion of mosquitos was proved to connote protection from malaria, and the crucial experiment of the younger Manson, who submitted to be bitten in England by a mosquito imported from Italy, and contracted malarial fever, not only proved the mosquito-malarial theory, but pointed out with precision the conditions necessary for success—a notable and far-reaching triumph in preventive medicine in which Englishmen bore a distinguished part.

The success which attended the researches into malaria was not long in bearing fruit. Drs. Walter Reed and James Carroll of the U.S.A. Army published in the year 1901 a paper in the journal of the American Public Health Association giving the results of some researches and experiments in the etiology of yellow fever. In brief, it was shown that, as in the case of malaria, the causal organism is a blood parasite, and that there is no known method by which the infection is transmitted from man to man except through the intervention of the mosquito. In this case, too, as in malaria, one species only of mosquito is effective *(Stegomyia fasciata)*. I will not tire you on this occasion by details of the discovery, which are of fascinating interest, but will content myself by quoting from the description of Surgeon-Major Gorgas, U.S.A., one crucial experiment which by its success added another triumph to man's "unconquerable mind."

By carefully-designed measures directed solely to the control of mosquito infection, the deaths in the months of April, May, June, July and August, the period of yellow fever prevalence, were reduced from the previously high figures, amounting, for example, in 1897 to 603, so that in 1901 they numbered three only, although the conditions as regards the non-immune were unfavourable, and infection was shown to be present by the continued appearance of new cases of fever.

**Bubonic Plague** affords a further example, showing the high importance of a study of the natural history of individual diseases.

The pandemic extension of plague which apparently started in 1894, when the disease reached Hong-Kong from Canton, and subsequently extended to the Indian Empire in 1896, arrested the attention of epidemiologists, and has disclosed very intricate inter-relations between human and animal infection, which are but dimly understood at present, but which bid fair to be solved by careful work on the comparative pathology of susceptible animals.

The susceptibility of the rat to infection is admitted on every hand, but the exact degree in which the rat is an "effective" agent in promoting outbreaks has been the subject of much controversy.

The most pressing danger to the British Islands culminated in the years 1900–1, by which time Egypt, Oporto, South and North America, and Australia had in turn become infected.

In the varying outbreaks the rat displayed the most varying degrees of "effectiveness" as an infective agent, leading to much difference of opinion. For example, in Australia it was clearly shown that not only the 1900 outbreak, but subsequent outbreaks, were rat introduced and rat spread; but in the Glasgow introduction of 1901, although the rat was an effective agent at first, it led only to a strictly limited outbreak of five cases. The careful search which was instituted in the neighbourhood showed that a considerable amount of rat-infection had taken place: at one time no less than forty out of sixty animals examined were found to be suffering from plague. Yet this rat-infection proved beyond this point quite "ineffective"; and Cardiff had a similar experience in the year 1900, when considerable infection amongst the rats in the dock warehouses proved ineffective in regard to human infection. Thus the rat theory became discredited. But the analogy of malaria and of yellow fever, in which a particular species alone is an "effective" agent in continuing the disease, suggests that similar conditions may hold in the case of plague.

Recent work on this disease points strongly to the truth of this supposition, and while acknowledging the influence of rat-infection in certain plague outbreaks, seeks to determine the causes for the varying efficiency of rat-infection in different outbreaks.

Captain W. G. Liston, I.M.S., in a paper read before the Bombay Natural History Society,[1] points out that two species of domestic rat exist there:—

1. The *Mus decumanus*—the common rat of Europe, living in drains and cellars.
2. The *Mus rattus*—the common rat of Bombay and the East generally, living in the roofs of houses, and even in trees.

And he states that plague may rage amongst Variety 1 without there being any great risk of communication to man, but that if the epizoötic disease occur amongst Variety 2 there is great risk of its spread amongst men.

He also states that the disappearance of plague from Europe was coincident with the invasion of that continent by the brown rat and the displacement of the black rat.

This suggestion agrees in effect with the observation of Cantlie[2] that the distribution of permanently endemic plague in India coincides with the geographical distribution of a specially susceptible sub-family of rats.

It corresponds also with the known facts in the epidemiology of malaria and of yellow fever, in which a particular species of mosquito is needed to act as an effective carrier of infection. To some such *ineffective* condition of the infected rat population must also, it would appear, be referred the continued immunity of Great Britain from serious plague invasion in spite of the recurrent introductions to which I have already referred

In a recent contribution to the epidemiology of plague, Mr. G. H. Hankin, Bacteriologist to the United Provinces and

[1] *Lancet*, 1905, i. 514; also *Times of India*, 26th Nov., 1904.

[2] *Tr. Epidem. Soc. Lond.*, 1896, xvi. 15.

to the Central Provinces, India, states as a result of personal observations:—

1. That no definite relation has been observed between intensity of plague (apart from its persistence) and badness of sanitation of dwellings in *epidemic* areas, though such conditions are generally found in *endemic* areas.

2. That where endemicity prevails it is probable that a latent stage of plague exists, during which neither human beings nor rats are affected.

3. That upon the introduction of plague into a district three periods occur—

(*a*) Spread of plague to some contacts.

(*b*) A period of quiescence, lasting from ten or twenty days to three or more months.

(*c*) A period of local activity, which may be indicated by the death of rats, preceding a human outbreak; or by simultaneous attack in rat and man; or by a purely human outbreak.

He considers that the long quiescent period between the first isolated group of cases (*a*) and the local activity (*c*) indicates not a simple contagion from man to man, but a deep-seated and perhaps complicated method of transmission. The agency of rats in spreading infection is considered to be of variable potency at different periods of an epidemic, and there is no apparent quantitative relation between man and rat mortality, nor are dejecta of man or of rat apparently important sources of infection. Hankin further considers that biting insects do not commonly act as direct intermediaries between man and man, or rat and man, and that the true view of the matter is probably this: that the "nidus" of the plague infection is some species of flea in which the microbe causes a slowly-developing infection, which at length becomes capable of transmitting the disease, and that in this insect the virus can retain or regain its virulence. These conclusions were supported by observations made in 1901 at Agra.

I have touched upon only a few instances in which the close study of the natural history of disease has proved to be all important. Much work and infinite thought is being expended upon kindred subjects in relation to typhoid and paratyphoid fever, in relation to human and bovine tuberculosis, and in other directions, all of supreme interest to the physician. Mr. J. B. Burke, in his *Origin of Life*, discusses still more recondite problems, and seeks to go back to the beginnings of life: a return, under different conditions, to the *de novo* researches of the last century.

Little by little man is wresting the truths of Nature from her jealous grasp and turning them to his advantage, but the way is steep, the progress slow, and steps have often to be retraced. The seeker after the great truths of Nature may well say with Newton, "I do not know what I may appear to the world, but to myself I seem to have been only like a boy playing on the seashore, and diverting myself in now and then finding a smoother pebble or a prettier shell than ordinary, whilst the great ocean of truth lay all undiscovered before me."

